# BLV-CoCoMo-qPCR: Quantitation of bovine leukemia virus proviral load using the CoCoMo algorithm

**DOI:** 10.1186/1742-4690-7-91

**Published:** 2010-11-02

**Authors:** Mayuko Jimba, Shin-nosuke Takeshima, Kazuhiro Matoba, Daiji Endoh, Yoko Aida

**Affiliations:** 1 Viral Infectious Diseases Unit, RIKEN, 2-1 Hirosawa, Wako, Saitama 351-0198, Japan; 2 Laboratory of Viral Infectious Diseases, Department of Medical Genome Sciences, Graduate School of Frontier Science, The University of Tokyo, Wako, Saitama 351-0198, Japan; 3 National Institute of Livestock and Grassland Science, 768 Senbonmatsu, Nasushiobara, Tochigi 329-2793, Japan; 4 School of Veterinary Medicine Department of Veterinary Medicine, Rakuno Gakuen University, 583 Midorimachi Bunkyodai Ebetsu, Hokkaido 069-8501 Japan

## Abstract

**Background:**

Bovine leukemia virus (BLV) is closely related to human T-cell leukemia virus (HTLV) and is the etiological agent of enzootic bovine leukosis, a disease characterized by a highly extended course that often involves persistent lymphocytosis and culminates in B-cell lymphomas. BLV provirus remains integrated in cellular genomes, even in the absence of detectable BLV antibodies. Therefore, to understand the mechanism of BLV-induced leukemogenesis and carry out the selection of BLV-infected animals, a detailed evaluation of changes in proviral load throughout the course of disease in BLV-infected cattle is required. The aim of this study was to develop a new quantitative real-time polymerase chain reaction (PCR) method using Coordination of Common Motifs (CoCoMo) primers to measure the proviral load of known and novel BLV variants in clinical animals.

**Results:**

Degenerate primers were designed from 52 individual BLV long terminal repeat (LTR) sequences identified from 356 BLV sequences in GenBank using the CoCoMo algorithm, which has been developed specifically for the detection of multiple virus species. Among 72 primer sets from 49 candidate primers, the most specific primer set was selected for detection of BLV LTR by melting curve analysis after real-time PCR amplification. An internal BLV TaqMan probe was used to enhance the specificity and sensitivity of the assay, and a parallel amplification of a single-copy host gene (the bovine leukocyte antigen *DRA *gene) was used to normalize genomic DNA. The assay is highly specific, sensitive, quantitative and reproducible, and was able to detect BLV in a number of samples that were negative using the previously developed nested PCR assay. The assay was also highly effective in detecting BLV in cattle from a range of international locations. Finally, this assay enabled us to demonstrate that proviral load correlates not only with BLV infection capacity as assessed by syncytium formation, but also with BLV disease progression.

**Conclusions:**

Using our newly developed BLV-CoCoMo-qPCR assay, we were able to detect a wide range of mutated BLV viruses. CoCoMo algorithm may be a useful tool to design degenerate primers for quantification of proviral load for other retroviruses including HTLV and human immunodeficiency virus type 1.

## Background

Many viruses mutate during evolution, which can lead to alterations in pathogenicity and epidemic outbreaks [1,2]. The development of molecular techniques, especially those applications based on the polymerase chain reaction (PCR), has revolutionized the diagnosis of viral infectious diseases [3,4]. Degenerate oligonucleotide primers, which allow the amplification of several possible mutated versions of a gene, have been successfully used for cDNA cloning and for the detection of sequences that are highly variable due to a high rate of mutation [5]. Degenerate primers are useful for the amplification of unknown genes, and also for the simultaneous amplification of similar, but not identical, genes [6]. The use of degenerate primers can significantly reduce the cost and time spent on viral detection. The "Coordination of Common Motifs" (CoCoMo) algorithm has been developed especially for the detection of multiple virus species (Endoh D, Mizutani T, Morikawa S, Hamaguchi I, Sakai K, Takizawa K, Osa Y, Asakawa M, Kon Y, Hayashi M: CoCoMo-Primers: a web server for designing degenerate primers for virus research, submitted). This program uses an extension of the COnsensus-DEgenerate Hybrid Oligonucleotide Primer (CodeHop) technique [7], which is based on multiple DNA sequence alignments using MAFFT multiple sequence alignment program [8]. The CoCoMo selects common gap tetranucleotide motifs (GTNM), which include codons from the target sequences. It then selects amplifiable sets of common GTNMs using a database-based method and constructs consensus oligonucleotides at the 5' end of each common amplifiable GTNM. The consensus degenerate sequence is then attached to the designed degenerate primers. Thus, the CoCoMo algorithm is very useful in the design of degenerate primers for highly degenerate sequences.

Bovine leukemia virus (BLV) is closely related to human T-cell leukemia virus types 1 and 2 (HTLV-1 and -2) and is the etiological agent of enzootic bovine leukosis (EBL), which is the most common neoplastic disease of cattle [9]. Infection with BLV can remain clinically silent, with cattle in an aleukemic state. It can also emerge as a persistent lymphocytosis (PL), characterized by an increased number of B lymphocytes, or more rarely, as a B-cell lymphoma in various lymph nodes after a long latent period [9].

In addition to the structural and enzymatic Gag, Pol, and Env proteins, BLV encodes at least two regulatory proteins, namely Tax and Rex, in the pX region located between the *env *gene and the 3' long terminal repeat (LTR) [9]. Moreover, BLV contains several other small open reading frames in the region between the *env *gene and the *tax/rex *genes in the pX region. These encode products designated as R3 and G4 [10]. BLV has two identical LTRs, which possess a U3 region, an unusually long R region, and a U5 region; these LTRs only exert efficient transcriptional promoter activity in cells productively infected with BLV [9]. BLV can integrate into dispersed sites within the host genome [11] and appears to be transcriptionally silent *in vivo *[12]. Indeed, transcription of the BLV genome in fresh tumor cells or in fresh peripheral blood mononuclear cells (PBMCs) from infected individuals is almost undetectable by conventional techniques [12,13]. *In situ *hybridization has revealed the expression of viral RNA at low levels in many cells, and at a high level in a few cells in populations of freshly isolated PBMCs from clinically normal BLV-infected animals [14]. It appears that BLV provirus remains integrated in cellular genomes, even in the absence of detectable BLV antibodies. Therefore, in addition to the routine diagnosis of BLV infection using conventional serological techniques such as the immunodiffusion test [15-18] and enzyme-linked immunosorbent assay (ELISA) [17-20], diagnostic BLV PCR techniques that aim to detect the integrated BLV proviral genome within the host genome are also commonly used [17-19,21-23]. However, real-time quantitative PCR for BLV provirus of all known variants has not been developed, largely due to differences in amplification efficiency caused by DNA sequence variations between clinical samples.

BLV infects cattle worldwide, imposing a severe economic impact on the dairy cattle industry [16-20,24-26]. Recent studies on the genetic variability of the BLV *env *gene have shown genetic variations among BLV isolates from different locations worldwide [24,27]. Therefore, in this study, we used the CoCoMo algorithm to design degenerate primers addressing BLV diversity and used these primers to develop a new quantitative real-time PCR method to measure the proviral load of all BLV variants. To normalize the viral genomic DNA, the BLV-CoCoMo-qPCR technique amplifies a single-copy host gene [bovine leukocyte antigen (*BoLA*)-*DRA *gene] in parallel with the viral genomic DNA. The assay is specific, sensitive, quantitative and reproducible, and is able to detect BLV strains from cattle worldwide, including those for which previous attempts at detection by nested PCR failed. Interestingly, we succeeded in confirming that the BLV copy number in PBMC clearly increased with disease progression.

## Results

### Principle of absolute quantification for determination of BLV proviral copy number

**Figure 1:**
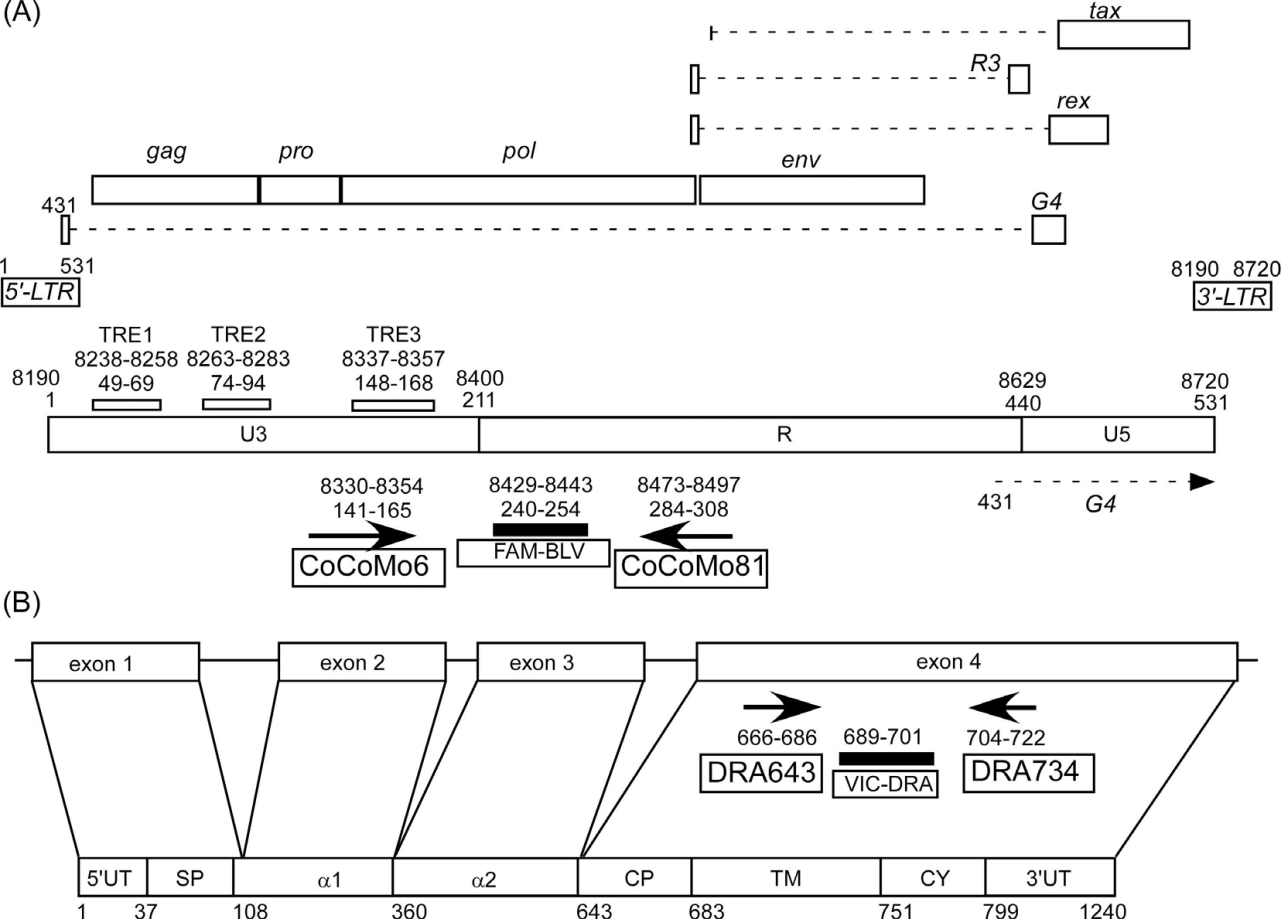
**The position, length and orientation of primers and probes used in the bovine leukemia virus (BLV)-CoCoMo-qPCR method**. Labeled arrows indicate the orientation and length of each primer. The black filled box indicates the probe annealing position. (A) The proviral structure of BLV in the BLV cell line FLK-BLV subclone pBLV913, complete genome [DDBJ: EF600696]. It contains two LTR regions at nucleotide positions 1-531 and 8190-8720. Lowercase labels indicate these LTR regions. The upper number shows the position of the 5' LTR and the lower number shows the position of the 3'LTR. Both LTRs include the U3, R and U5 regions. A triplicate 21-bp motif known as the Tax-responsive element (TRE) is present in the U3 region of the 5' LTR. The target region for amplification was in the U3 and R region, and the TaqMan probe for detecting the PCR product was from the R region. (B) The schematic outline of the bovine major histocompatibility complex (*BoLA*)*-DRA *gene (upper) and its cDNA clone MR1 [DDBJ: D37956] (lower). Exons are shown as open boxes. The numbers indicate the numbering of the nucleotide sequence of MR1. 5'UT, 5'-untranslated region; SP, signal sequence; α1, first domain; α 2, second domain; CP, connecting peptide; TM, transmembrane domain; CY, cytoplasmic domain; 3'UT, 3-untranslated region. The target regions for amplification and for binding of the TaqMan probe to detect the PCR product are in exon 4.

To determine the absolute copy number of BLV provirus, we selected the LTR region as a target sequence for PCR amplification (Figure 1A). In designing the assay, we took into account the fact that two LTRs will be detected for each individual BLV genome (see equation below). To normalize genomic DNA input, the assay also included a parallel amplification of the single-copy *BoLA-DRA *gene (Figure 1B). The number of proviral copies per 100,000 cells is calculated according to the following equation:

$$\begin{array}{l} \text{BLV provirus load}\\ =\text{BLV provirus copy number}/\text{diploid cell number}\times \text{1}00,000\text{ cells}\\ =\left(\text{BLV LTR copy number}/\text{2}\right)/(\text{BoLA}-\text{DRAcopy number}/\text{2})\times \text{1}00,000\text{ cells }\left(\text{A}\right) \end{array}$$

### Use of the CoCoMo algorithm to construct a primer set with the ability to amplify all BLV strains

**Figure 2:**
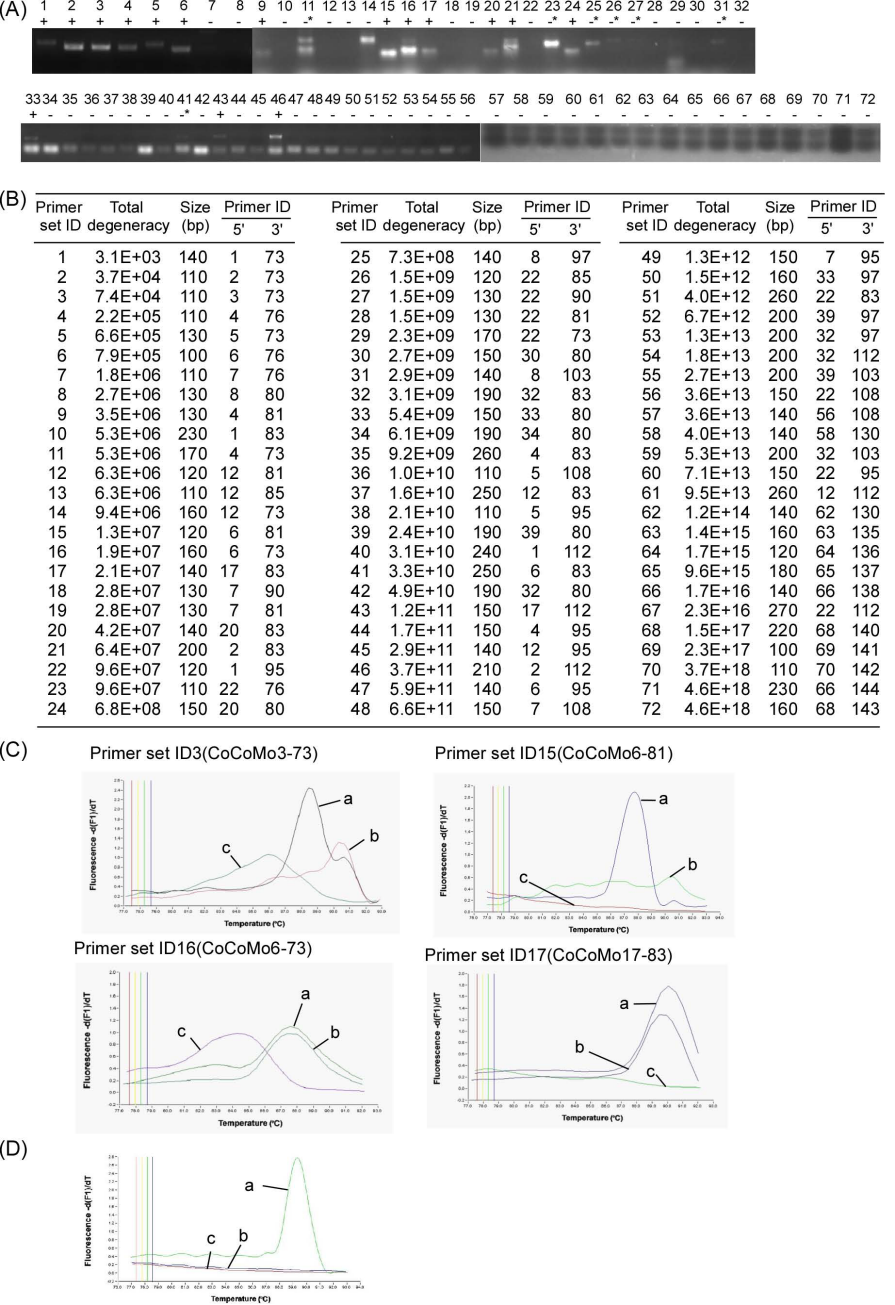
**Selection of the primer set for amplification of the BLV LTR region**. (A) Touch-down PCR was performed using 72 primer sets with 49 primers designed by the CoCoMo program as shown in Table 1. PCR products were detected by electrophoresis on a 3% agarose gel. Lanes 1-72, 1-72 primer set ID; +, results positive for PCR product; -, negative results for same. *, designates PCR products that were detected but for which the amplicon sizes differed from the predicted size. (B) Summary of results shown in (A). Primer set IDs are arranged according to the degeneracy of the primer set and size of the PCR products. (C) The 4 representative melting curves with 16 primer sets of: BLV-infected BLSC-KU-17 cells (a), BLV-free normal cattle cells (b), and reagent-only as negative control (c). The specificity of the 16 selected primer sets was checked by melting curve analysis. Each PCR amplification was followed by gradual product melting at up to 95°C. (D) The optimization of PCR amplification with primer set ID 15 (CoCoMo 6 and 81). The melting curve of PCR products from BLV-infected BLSC-KU-17 cells (a), the BLV-free normal cattle Ns118 (b), and reagent-only as negative control (c).

To amplify all BLV variants, primers targeting the BLV LTR region were constructed using the modified CoCoMo algorithm, which was developed to design PCR primers capable of amplifying multiple strains of virus. We collected 356 BLV nucleotide sequences from GenBank (on 30^th ^April, 2009). From these BLV sequences, 102 LTR sequences were selected according to GenBank annotations (Additional file 1). From the LTR sequences, we selected 85 sequences that were large enough to determine homologies and assigned the sequences to major BLV LTR groups based on homology using a graphical approach with Pajek graphical software (Additional file 2). Fifty two of these sequences were selected for primer design (Additional file 3). The target sequences were subjected to a BLV LTR modified version of the CoCoMo-primer-design algorithm, which was developed for designing degenerate primers to detect multiple strains of virus. Using these sequences as templates, a total of 72 primer sets (Figure 2B) with 49 candidate primers (Table 1) were designed.

**Table 1:** Primer sequences for amplification of BLV LTR candidate regions by the Coordination of Common Motif (CoCoMo) algorithm

Primer ID	Sequence	**Primer annealing position in the BLV LTR sequence**^**1**^	
		
		3'LTR	5'LTR
1	ACCTGYYGWKAAAYTAATAMAATGC	162-186	8351-8375
2	CYDKYSRGYTARCGGCRCCAGAAGC	192-216	8381-8405
3	GSCCYDKYSRGYTARCGGCRCCAGA	189-213	8378-8402
4	VRRAAWHYMMNMYCYKDAGCTGCTG	132-156	8321-8345
5	KDDWAAHTWAWWMAAWKSCGGCCCT	169-193	8358-8382
6	MNMYCYKDRSYKSYKSAYYTCACCT	141-165	8330-8354
7	YYSVRRAAWHYMMNMYCYKDAGCTG	129-153	8318-8342
8	GCTCCCGAGRCCTTCTGGTCGGCTA	266-290	8455-8479
12	NMYCYKDRSYKSYKSAYYTCACCTG	142-166	8331-8355
17	SGKYCYGAGYYYKCTTGCTCCCGAG	250-274	8439-8463
20	YSGKYCYGAGYYYKCTTGCTCCCGA	249-273	8438-8462
22	HVVRRWMHHYMMNMYSHKNWGCTGC	130-154	8319-8343
30	YYYSGKYCYGAGYYYKCTTGCTCCC	247-271	8436-8460
32	SGSMVCMRRARSBRYTCTYYTCCTG	204-228	8393-8417
33	YYYYSGKYCYGAGYYYKCTTGCTCC	246-270	8435-8459
34	VCMRRARSBRYTCTYYTCCTGAGAC	208-232	8397-8421
39	GSMVCMRRARSBRYTCTYYTCCTGA	205-229	8394-8418
56	MNMYMYDNVSYKVBBBRYYKCACCT	141-165	8330-8354
58	YSBRRGBYBKYTYKCDSCNGAGACC	253-277	8442-8466
62	BYSBRRGBYBKYTYKCDSCNGAGAC	323-347	8441-8465
63	YYYYBGBYYYSWGHYYBCKYGCTCC	246-270	8587-8611
64	VRDNYHHNHYYYBNRKYYBYTGACC	354-378	8324-8348
65	HVVNVHVNHHVVNVNSNKNWGMYGS	43-67, 68-92, 130-154	8232-56, 8257-81, 8319-43
66	NNHHDHBHRWDMMAHNSMBDSMSYK	124-148, 169-193, 170-194	8313-37, 8358-82, 8359-83
68	BNNVBBHVNVHNYYYBNYHVMYBHS	26-50, 91-115, 247-271	8215-39, 8280-8304, 8436-60
69	NVMNBNNHHVDNHWMHYSMBRMSCT	123-147, 128-152, 211-235	8312-36, 8317-41, 8400-24
70	NNNBBHVBVNNHNBBRHYYBTCTCC	202-226, 360-384, 375-399	8391-8415, 8549-73, 8564-88
73	TGGTCTCHGCYGAGARCCNCCCTCC	325-349	8514-8538
76	GCCGACCAGAAGGYCTCGGGAGCAA	264-288	8453-8477
80	SSSRKKBVVRVSCMRRMSSCCTTGG	421-445	8610-8634
81	TACCTGMCSSCTKSCGGATAGCCGA	284-308	8473-8497
83	KKBVVRVSCMRRMSSCCTTGGAGCG	417-441	8606-8630
85	GMCSSCTKSCGGATAGCCGACCAGA	279-303	8468-8492
90	CCTGMCSSCTKSCGGATAGCCGACC	282-306	8471-8495
95	YYYMMVMVBBKKNBTDKCCTTACCT	304-328	8493-8517
97	RMVVRDVBVVGVBDSMVRSCCWKRS	421-445, 429-453	8610-8634, 8618-8642
103	VMVVVDRVNVSSVDKVMRVSCYWGR	421-445, 430-454	8610-8634, 8619-8643
108	YYMMVMVBBKKNBTDKCCTTACCTG	303-327	8492-8516,
112	VVVRRNBSVRRBBVVRVSCCMKWSG	421-445, 428-452	8610-8634, 8617-8641
130	NKNVVRVSCVVVVVVVSWKRGAGCG	417-441, 484-508	8606-8630, 8673-8697
135	NNVVNDRVNVBNNDKNNNNNBHNND	4-28, 90-114, 105-129, etc	8610-8634, 8619-8643, etc
136	BHYYYBNSSSVHKVSRGRKMGCCGA	284-308, 495-519	8473-8497, 8684-8708
137	DRRRSYHVSVRDRSTCDSDRCCGAG	247-271, 336-360	8436-8460, 8525-8549
138	WWVVDSHYSSVKKSSKSWYWGCCGA	284-308, 337-861	8473-8497, 8526-8550
140	NHNNNBBBSSVVTRGWSKSHGCCGA	337-361, 495-519	8526-8550, 8684-8708
141	NRRRVBHVVVRDRSYYNSDRCCGAG	247-271, 336-360	8436-8460, 8525-8549
142	NHNNNBBBSSVNYDSWSBBNGCCGA	337-361, 495-519	8526-8550, 8684-8708
143	VMVVVNDNNVSSVDDVMVVVCYWGR	279-303, 421-445, 430-454	8468-8492, 8610-34, 8619-43
144	VVVRRNNVVRDBBVVVVBSSMKWSG	378-402, 421-445, 428-452	8567-8591, 8610-34, 8617-41

^1^Numbers indicate the position in the nucleotide sequence of the FLK-BLV subclone pBLV913 [DDBJ: EF600696].

### Selection of the primer set and probe for amplification of the BLV LTR region

To determine whether the CoCoMo primer sets amplified the BLV LTR region, touch-down PCR was performed with 72 candidate primer sets (Figure 2B) using genomic DNA extracted from BLV-infected BLSC-KU-17 cells. As shown in Figure 2A, we identified 16 sets of primers, 1-6, 9, 15-17, 20, 21, 24, 33, 43 and 46, which successfully amplified the BLV LTR region.

The specificity of the 16 selected primer sets was evaluated by melting-curve analysis of amplification using genomic DNA extracted from BLSC-KU-17 cells or PBMCs from BLV-free normal cattle Ns118, with reagent-only as the negative control. Figure 2C shows the four typical melting-curves. Amplicons consisting of a single PCR product with a single melting temperature exhibited a single peak, while amplicons consisting of two or more products exhibited multiple peaks. The amplicon generated using primer set ID15 of CoCoMo 6 and CoCoMo 81 had a single melting temperature using BLSC-KU-17 genomic DNA. Using these primers, no amplicons were generated using genomic DNA from PBMCs in BLV-free normal cattle Ns118 or using the reagent-only control. In contrast, other primer sets, such as ID3, ID16 and ID17 generated amplicons from genomic DNA extracted from PBMCs from BLV-free normal cattle Ns118 or in reagent only, as well as from genomic DNA extracted from BLSC-KU-17 cells. Therefore, we proceeded to optimize the amplification conditions using primer sets CoCoMo 6 and CoCoMo 81, which were the best pair for the detection of the BLV LTR region (Figure 2D). Under these optimized conditions, amplification melting-curve analysis using genomic DNAs extracted from 56 BLV-infected cattle and from 3 BLV-free normal cattle showed the same patterns as seen in BLSC-KU-17 cells and BLV-free normal cattle Ns118 (data not shown).

The internal BLV TaqMan probe was constructed from a region of low variability located between positions corresponding to the CoCoMo 6 and CoCoMo 81 primers in the LTR regions of the BLV genome (Figure 1), and was labeled with carboxyfluorescein (FAM) dye, non-fluorescent quencher (NFQ) and minor groove binder (MGB) probe for enhancing the probe melting temperature. The probe was designated as FAM-BLV.

**Figure 3:**
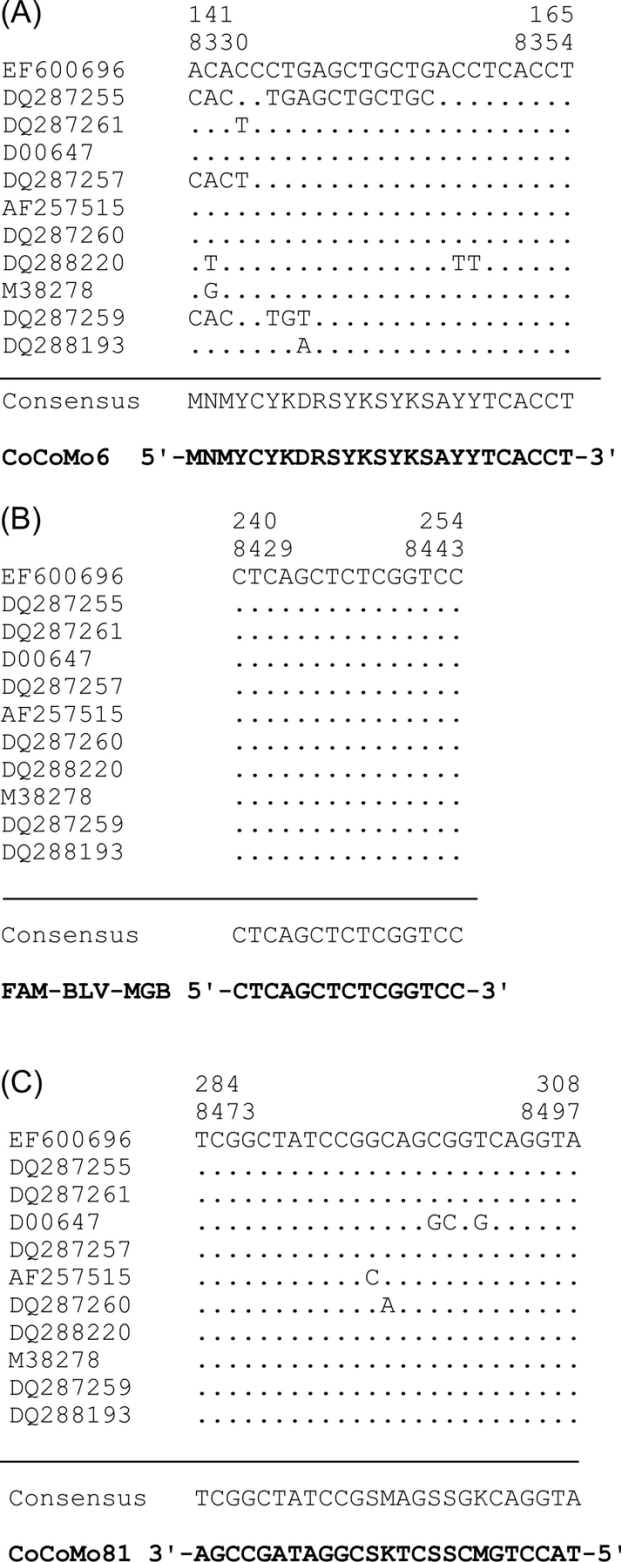
**Sequence alignment of annealing positions of the CoCoMo 6 primer (A), FAM-BLV-MGB probe (B) and CoCoMo 81 primer (C) in the 52 BLV LTR sequences**. The sequence alignment used 52 sequences from GenBank that were integrated into a total of 11 sequences, including 8 individual sequences for the CoCoMo 6 primer and 4 individual sequences for the CoCoMo81 primer. Accession numbers for the representative sequences are indicated in the left column. Numbers indicate the numbering of the nucleotide sequence of the FLK-BLV subclone pBLV913 [DDBJ: EF600696]. The upper number shows the position of the 5' LTR and the lower number shows the position of the 3' LTR.

Alignments of the sequences corresponding to the primer and probe regions from the 52 BLV LTR sequences taken from GenBank are shown in Figure 3. Based on this comparison, out of the 52 sequences, 8 individual sequences corresponding to CoCoMo 6 primer and 4 individual sequences for CoCoMo 81 primer could be arranged. The alignment demonstrated that although the sequences in the probe region were sufficiently conserved to allow alignment of the BLV variants, the sequences corresponding to the CoCoMo 6 and CoCoMo 81 primers exhibited a low degree of similarity.

### Construction of the primer set and probe for quantification of the BoLA-DRA gene

For normalization of the genomic DNA used as the PCR template, we designed primers and a probe for quantification of the *BoLA-DRA *gene (Figure 1). We obtained sequences from an MR1 cDNA clone [DDBJ: No. D37956] and selected the exon 4 region of the *BoLA-DRA *gene as the target for amplification. We designed the amplification primer set DRA643 and DRA734 and the internal BoLA-DRA TaqMan probe using the Primer Express 3.0 (Applied Biosystems, Tokyo, Japan). The probe was labeled with VIC dye, NFQ and an MGB probe for enhancing the probe melting temperature, and was designated as VIC-DRA.

### Quantification of plasmid DNA copy number to create standard curves for absolute quantitative PCR

To obtain standards for quantification of BLV proviral DNA and cellular DNA, pBLV-LTR/SK, which includes a full-length LTR of BLV, and pBoLA-DRA/SK, which includes a full-length bovine *DRA *gene, were prepared at 103.1 ng/μl (pBLV-LTR^conc^) and 125.0 ng/μl (pBoLA-DRA^conc^), respectively. The copy numbers of these plasmids were calculated by the serial dilution method: each plasmid was diluted 10-fold, and the target DNA was detected by nested PCR. For example, at a 10^-11 ^dilution of pBLV-LTR^conc^, PCR amplification failed to detect any PCR product, including the BLV LTR. The PCR reaction was then replicated 10 times at the 10^-11 ^dilution, and the success rate was found to be 5/10. This result showed that 5 of 10 PCR solutions did not contain the LTR gene, expressed in equation form as: f (x = 0) = 5/10. Finally, the average copy number of the target gene (λ) was calculated as -log_e _(5/10) = 0.231 corresponding to a copy number for pBLV-LTR^conc ^of 2.31 × 10^10^/μl. Using the same strategy, the copy number of pBoLA-DRA^conc ^was determined to be 2.54 × 10^10^/μl. For confirmation of the reliability of estimated copy numbers, we also calculated draft copy numbers from the DNA weight and obtained a very similar result (2.35 × 10^10 ^for pBLV-LTR^conc^, and 2.85 × 10^10 ^for pBoLA-DRA^conc^).

### Final procedure for the optimization of BLV-CoCoMo-qPCR

To construct the standard curve, the following dilutions of pBLV-LTR^conc ^and pBoLA-DRA^conc ^were created: 0.1 copy/μl, 1 copy/μl, 1,000 copies/μl and 1,000,000 copies/μl. A 168-bp amplicon from the BLV LTR region was amplified in a total volume of 20 μl of 1 × TaqMan Gene Expression Master Mix containing 500 nM CoCoMo 6 primer, 50 nM CoCoMo 81 primer, 150 nM FAM-BLV probe (5'-FAM-CTCAGCTCTCGGTCC-NFQ-MGB-3'), and 30 ng of template DNA. In addition, a 57-bp amplicon of the *BoLA-DRA *region was amplified in a total volume of 20 μl of 1 × TaqMan Gene Expression Master Mix containing 50 nM of DRA643 primer (5'-CCCAGAGACCACAGAGAATGC-3'), 50 nM of DRA734 primer (5'-CCCACCAGAGCCACAATCA-3'), 150 nM of VIC-DRA probe (5'-VIC-TGTGTGCCCTGGGC-NFQ-MGB 3'), and 30 ng of template DNA. PCR amplification was performed with the ABI 7500 Fast Real-time PCR system according to the following program: Uracil-DNA Glycosylase (UDG) enzyme activation at 50°C for 2 min followed by AmpliTaq Gold Ultra Pure (UP) enzyme activation at 95°C for 10 min, and then 85 cycles of 15 sec at 95°C and 1 min at 60°C. Copy numbers obtained for the BLV LTR and *BoLA-DRA *were used to calculate BLV proviral load per 100,000 cells, as shown in Equation (A).

### Reproducibility of BLV-CoCoMo-qPCR

The intra- and inter-assay reproducibility of BLV-CoCoMo-qPCR for determination of BLV proviral copy number was evaluated using aliquots of genomic DNA extracted from blood samples from seven BLV-infected cattle (Table 2). For determination of intra-assay reproducibility, we examined triplicate PCR amplifications from each sample, with the assay being repeated three times. A total of 21 examinations were performed, and the intra-assay coefficient of variance (CV) ranged from 0% to 20.5% (mean 8.6%). For determination of inter-assay reproducibility, we performed three independent experiments for each sample. The values for the inter-assay CV for BLV proviral copy number per 100,000 cells ranged from 5.5% to 19.8% (mean 12.7%). These results clearly demonstrated that this assay has good intra- and inter-assay reproducibility.

**Table 2:** Intra- and inter-assay reproducibility of BLV-CoCoMo-qPCR

**No.**	**Proviral load^1^**			**Intra-assay**^**2**^			**Inter-assay**^**3**^
			
	**Exp.1**	**Exp 2**	**Exp 3**	**Exp.1**	**Exp.2**	**Exp.3**	**Exp.1~3**
			
Ns105	1998 ± 385	2107 ± 296	1581 ± 150	17.9	14.1	9.5	14.6
Ns209	3951 ± 691	3751 ± 529	3049 ± 150	17.5	14.1	4.9	13.2
Ns126	20388 ± 222	23484 ± 1854	28375 ± 1381	1.1	7.9	4.9	16.7
Ns226	30155 ± 6184	27247 ± 1454	34954 ± 3021	20.5	5.3	8.6	12.6
Ns120	57236 ± 6127	59375 ± 3195	53225 ± 2514	10.7	5.4	4.7	5.5
Ns107	90947 ± 0	73002 ± 3228	61388 ± 4779	0.0	4.4	7.8	19.8
Ns112	87377 ± 7434	94934 ± 5891	84667 ±5763	8.5	6.2	6.8	6.0

^1^Values represent the mean ± standard deviation (SD) of BLV proviral copy numbers in 10^5 ^cells from triplicate PCR amplifications from each sample.

^2^Intra-CV: Coefficient of variation between each sample.

^3^Inter-CV: Coefficient of variation between each experiment.

### Evaluation of the specificity of BLV-CoCoMo-qPCR primers using various retroviruses

**Figure 4:**
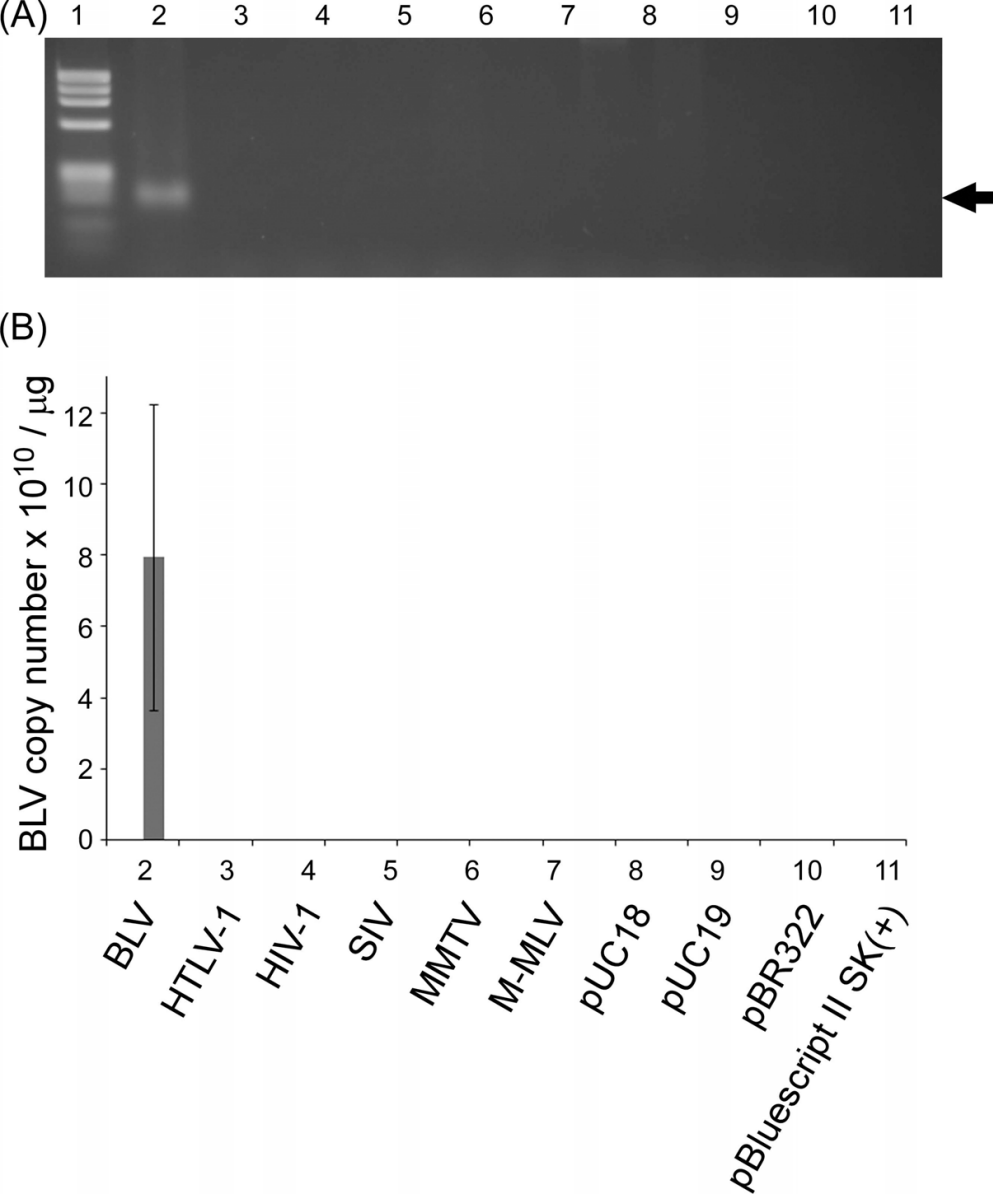
**Evaluation of the specificity of the BLV-CoCoMo-qPCR primers**. (A) Real-time PCR using the CoCoMo 6 and CoCoMo 81 primers from the BLV-CoCoMo-qPCR was performed using 0.3 ng of the following infectious molecular clones: BLV (pBLV-IF, lane 2); HTLV-1 (pK30, lane 3); HIV-1 (pNL4-3, lane 4); SIV (pSIVmac239/WT, lane 5); MMTV (hybrid MMTV, lane 6); M-MLV (pL-4, lane 7); and the plasmids pUC18 (lane 8), pUC19 (lane 9), pBR322 (lane 10), and pBluescript SK(+) (lane 11). PCR products were subjected to 3% agarose gel electrophoresis. Lane 1, DNA marker Φ × 174-*Hae *III digest. A PCR product 168 bp in length is indicated by an arrow. (B) The number of BLV provirus copies in 1 μg of DNA from each DNA sample is indicated by lowercase. Values represent the mean ± standard deviation (SD) of the results of three independent experiments.

The specificity of BLV-CoCoMo-qPCR primers was tested using various retroviral molecular clones, including BLV, HTLV-1, human immunodeficiency virus type 1 (HIV-1), simian immunodeficiency virus (SIV), mouse mammary tumor virus (MMTV), Molony murine leukemia virus (M-MLV), and a range of plasmids including pUC18, pUC19, pBR322, and pBluescript II SK (+). For real-time PCR, CoCoMo 6 and CoCoMo 81 primers were used with 0.3 ng of each plasmid, and the products were analyzed by 3% agarose-gel electrophoresis. A single PCR product, 168-bp in length, was observed only for the BLV infectious molecular clone (Figure 4A), with a copy number of 7.9 × 10^10^/μg ± 4.3 × 10^10^/μg (Figure 4B). No amplicons were detected for any of the other plasmids. These results strongly indicate that BLV-CoCoMo-qPCR primers specifically amplify the BLV LTR without amplifying the LTRs of other retroviruses.

### Evaluation of the sensitivity of BLV-CoCoMo-qPCR compared with nested PCR

**Figure 5:**
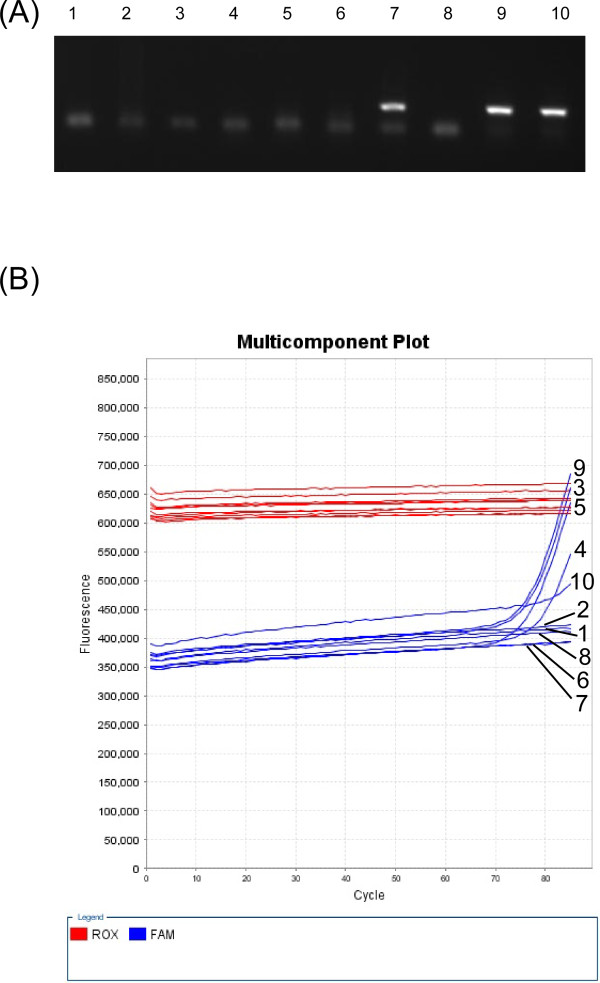
**Comparison of the sensitivity of BLV-CoCoMo-qPCR and nested PCR**. Ten samples containing 0.7 copies of pBLV-LTR/SK were amplified by nested PCR (A) and real-time PCR with the CoCoMo 6 and CoCoMo 81 primer set (B). The 168-bp band was used to detect BLV LTR amplicons (A). Carboxy-X-rhodamine (ROX) intensities were used for corrections of tube differences, and carboxyfluorescein (FAM) intensities were used to detect BLV LTR amplicons (B).

To determine the sensitivity of BLV-CoCoMo-qPCR, 20 solutions, each containing 0.7 copies of pBLV-LTR/SK, were amplified by nested PCR and real-time PCR using the CoCoMo 6 and CoCoMo 81 primer set (Figure 5). Three out of ten nested PCR amplifications were positive, and the copy number was estimated to be 0.36. For real-time PCR using the CoCoMo 6 and CoCoMo 81 primer set, five out of ten PCR amplifications were positive, and the copy number was estimated to be 0.69. This result showed that the sensitivity of BLV-CoCoMo-qPCR was 1.9-fold greater than that of nested PCR.

### Comparison of BLV-CoCoMo-qPCR and Serial dilution nested PCR

**Figure 6:**
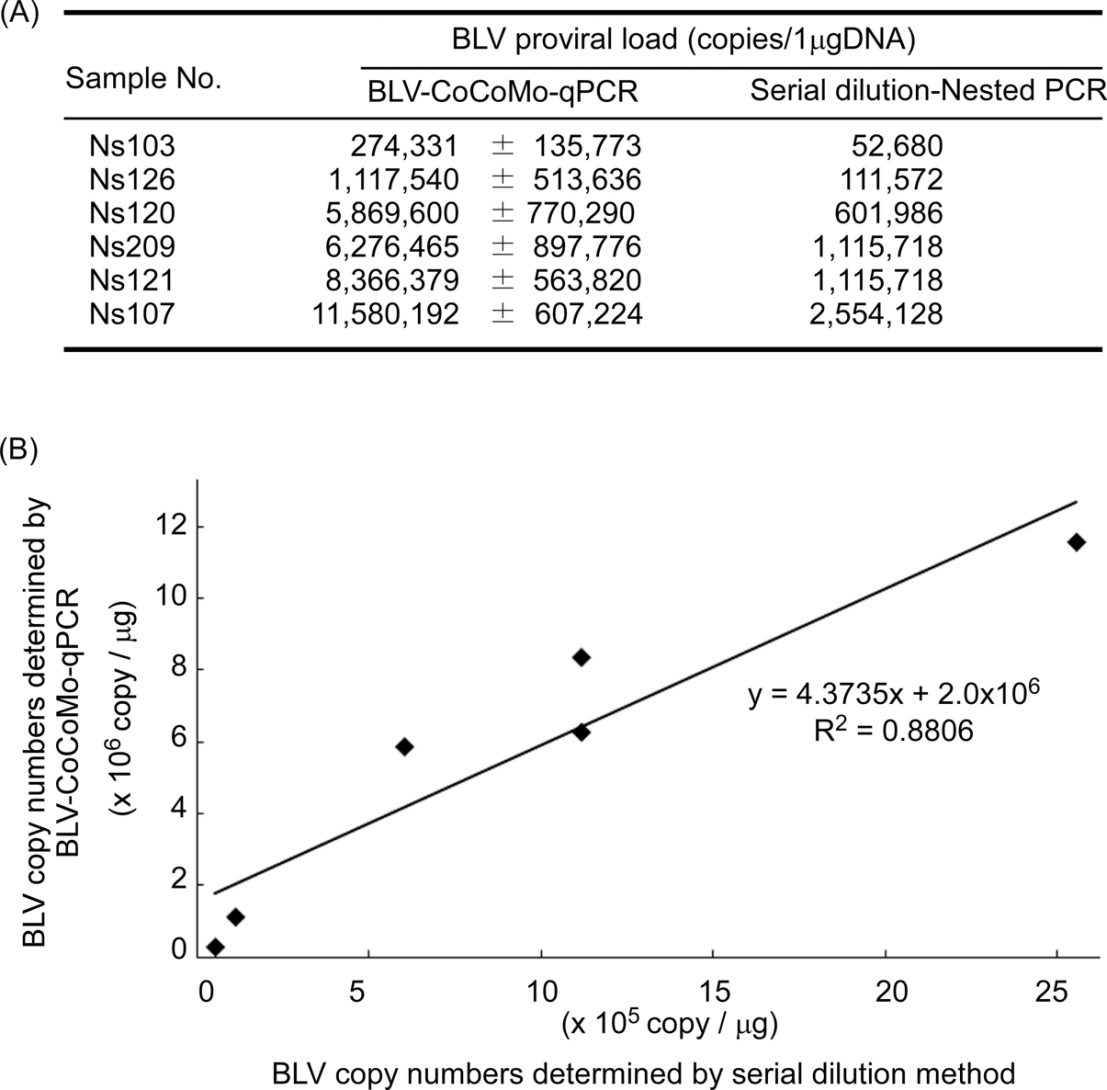
**Correlation between proviral load calculated by BLV-CoCoMo-qPCR and serial dilution nested PCR**. (A) BLV proviral copy numbers for 1 μg of genomic DNA from 6 BLV-infected cattle were determined by BLV-CoCoMo-qPCR and serial dilution-nested PCR. For serial dilution-nested PCR, the 6 genomic DNAs were analyzed by serial tenfold dilution and subjected to nested PCR for detection of the BLV LTR gene. Nested PCR reactions were repeated 10 times and proviral load was calculated according to a Poisson distribution model as shown in Methods. Values represent the mean ± standard deviation (SD) of results from four independent experiments. (B) Scatter chart is indicated the correlation between BLV copy numbers which were determined by BLV-CoCoMo-qPCR and by serial dilution-nested PCR.

The serial dilution method is effective for quantifying the copy number of a target gene. The BLV proviral copy number per 1 μg of genomic DNA was calculated for five BLV-infected cattle by serial dilution-nested PCR and real-time PCR with the CoCoMo 6 and CoCoMo 81 primer set (Figure 6A). The BLV proviral copy number obtained by both methods was confirmed by regression analysis: the square of the correlation coefficient (R^2^) was 0.8806 (Figure 6B), indicating that the copy number obtained by real-time PCR with the CoCoMo primers correlated with that obtained by serial dilution-nested PCR. Thus, it appears that real-time PCR with the CoCoMo 6 and CoCoMo 81 primer set can be used to obtain the copy number of BLV provirus from a clinical sample.

### Correlation of BLV-CoCoMo-qPCR and syncytium formation assay

**Figure 7:**
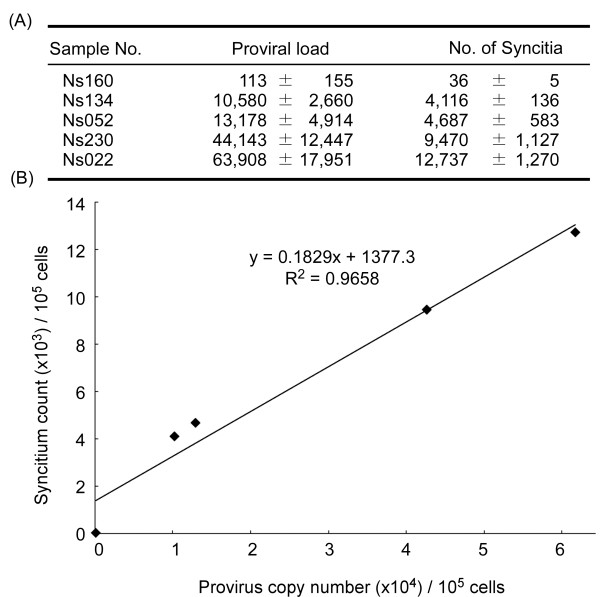
**Correlation between proviral load calculated by BLV-CoCoMo-qPCR and syncytium formation**. (A) Using BLV-CoCoMo-qPCR, the proviral loads from five BLV-infected cattle were calculated and shown as provirus copy number per 1 × 10^5 ^cells. A syncytium formation assay using CC81 indicator cells was used to count the number of syncytia per 1 × 10^5 ^peripheral blood mononuclear cells (PBMCs) from five BLV-infected cattle. Values represent the mean ± standard deviation (SD) of results from three samples. (B) Scatter chart is indicated the correlation between BLV copy numbers which were determined by BLV-CoCoMo-qPCR and the number of syncytia.

To test whether the BLV proviral copy number correlates with the capacity for infection with BLV, BLV-CoCoMo-qPCR and a syncytium formation assay were conducted on samples from five BLV-infected cattle. We evaluated the capacity for transmission of BLV by coculturing 1 × 10^5 ^PBMCs from five BLV-infected cattle with inducer CC81 cells for three days and comparing proviral copy numbers with 1 × 10^5 ^cells from the same cattle (Figure 7A). Proviral copy numbers ranged from 113 to 63,908 copies per 10^5 ^cells, and syncytium numbers ranged from 36 to 12,737 per 10^5 ^PBMCs. Regression analysis for these samples revealed that the level of provirus load positively correlated with the number of syncytia (R^2 ^= 0.9658), as shown in Figure 7B.

### BLV provirus detection in cattle from different geographic locations by BLV-CoCoMo-qPCR and nested PCR

BLV-CoCoMo-qPCR has the potential ability to detect various BLV strains, both known and unknown, because degenerate primers are capable of detecting highly degenerate sequences. In the experiments described above, we found that the sensitivity of BLV-CoCoMo-qPCR was greater than that of nested PCR. Therefore, we examined whether BLV-CoCoMo-qPCR can detect BLV provirus in cattle from different geographic locations worldwide. We tested 54 cattle from one farm in Japan, 15 cattle from two farms in Peru, 60 cattle from four farms in Bolivia, 32 cattle from three farms in Chile and 5 cattle from one farm in the U.S.A., and compared the results obtained by BLV-CoCoMo-qPCR with the results obtained by nested PCR (Table 3). The amplification of BLV LTR by the two methods divided the 166 cattle into three groups. The first group of cattle (n = 107) was positive for BLV LTR by both methods (50 in Japan, 7 in Peru, 27 in Bolivia, 18 in Chile, and 5 in U. S. A.). The second group of cattle (n = 50) was negative for BLV LTR by both methods (2 in Japan, 7 in Peru, 28 in Bolivia, and 13 in Chile). The third group of cattle (n = 9) was positive by BLV-CoCoMo-qPCR but negative by nested PCR (2 in Japan, 1 in Peru, 5 in Bolivia, and 1 in Chile). Interestingly, none of the cattle were negative by BLV-CoCoMo-qPCR but positive by nested PCR. Thus, the nested PCR and BLV-CoCoMo-qPCR methods gave the same result for 94.6% of the cattle tested, but for 5.4% of the cattle, only the BLV-CoCoMo-qPCR was able to detect BLV provirus. These results clearly showed that the sensitivity of BLV-CoCoMo-qPCR was higher than that of nested PCR.

**Table 3:** Comparison of BLV detection by BLV-CoCoMo-qPCR and nested PCR in cattle from Japan, Peru, Bolivia, Chile and the U.S.A.

		Results of BLV detection by BLV-CoCoMo-qPCR/nested PCR								
		
Country		**+/+**^1^		-/-		+/-		-/+		
						
		n^2^	%	n	%	n	%	n	%	Total number
Japan	A	50	92.6	2	3.7	2	3.7	0	0	54
Peru	A	7	77.8	2	22.2	0	0	0	0	9
	B	0	0	5	83.3	1	16.7	0	0	6
Bolivia	A	2	40.0	2	40.0	1	20.0	0	0	5
	B	4	66.7	2	33.3	0	0	0	0	6
	C	5	35.7	8	57.1	1	7.1	0	0	14
	D	16	45.7	16	45.7	3	8.6	0	0	35
Chile	A	11	68.8	4	25.0	1	6.2	0	0	16
	B	0	0	6	100.0	0	0	0	0	6
	C	7	70.0	3	30.0	0	0	0	0	10
USA	A	5	100.0	0	0	0	0	0	0	5

^1^+, positive for detection of BLV; -, negative for detection of BLV.

^2^n, number of samples with identical decisions based on two methods.

**Figure 8:**
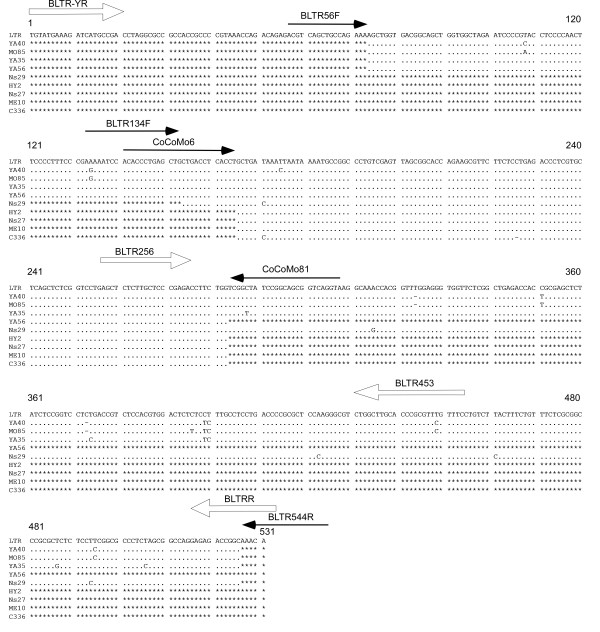
**Alignment of BLV LTR nucleotide sequences in samples that were positive by BLV-CoCoMo-qPCR but negative by nested PCR**. BLV LTR sequences from 9 BLV-infected cattle were amplified by PCR using three primer pairs, BLTR56F and CoCoMo81, BLTR134F and BLTR544R, and CoCoMo6 and CoCoMo81, followed by sequencing of the PCR products. Closed arrows indicate the position, orientation and length of these primers. The LTR sequences at nucleotide positions 74-283 and 154-526 from YA40, MO85 and YA35 were amplified using two primer pairs BLTR56F and CoCoMo81, and BLTR134F and BLTR544R, respectively. The LTR sequence at nucleotide positions 74-283 from YA56 was amplified by primer pair BLTR56F and CoCoMo81. The LTR sequences at nucleotide positions 166-283 from HY2, Ns27, ME10 and C336 were amplified by the primer pair CoCoMo6 and CoCoMo81. The LTR sequence at nucleotide positions 154-526 from Ns29 was amplified by primer pair BLTR134F and BLTR544R. Open arrows indicate the position, orientation and length of first primer pair, BLTR-YR and BLTRR, and the second primer pair, BLTR256 and BLTR453, for nested PCR. The numbers at the top of the sequences indicate the terminal bases according to the nucleotide sequence of the FLK-BLV subclone pBLV913 [DDBJ: EF600696]. Conserved sequences are indicated by a dot (.), deletions are indicated by a hyphen (-). A lack of sequence information is indicated by the symbol (*).

As shown in Table 3, we detected several samples that were positive by BLV-CoCoMo-qPCR, but negative by nested PCR. To confirm that these samples were infected with BLV, and to investigate why these samples were not detected by nested PCR, we sequenced the LTR region of nine samples from this group: YA40, MO85, YA35, YA56 and ME10 from Bolivia, HY2 from Peru, C336 from Chile, and Ns27 and Ns29 from Japan. We were able to detect BLV LTR sequences in all nine samples (Figure 8), thus confirming the high specificity of BLV-CoCoMo-qPCR. In two of the nine samples, we identified mismatch sequences at the annealing region for the primer BLTR453, which was used for amplification of the LTR in nested PCR. This is a possible explanation for why the nested PCR failed to detect the BLV provirus.

### Correlation analysis of disease progression and BLV proviral load

**Figure 9:**
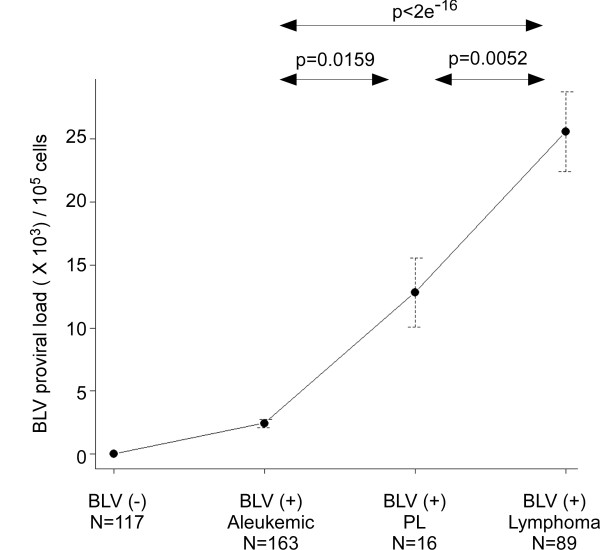
**Increased proviral load correlates with disease progression in BLV-induced enzootic bovine leukosis (EBL)**. The proviral load was calculated for 385 cattle by BLV-CoCoMo-qPCR. The cattle were classified into four disease stages according to diagnosis based on previously established criteria [41], the genomic integration of BLV, and the detection of antibodies to BLV: 117 BLV-negative cattle (BLV-); 163 BLV-infected cattle that were clinically and hematologically normal (aleukemic); 16 clinically normal, BLV-infected cattle with persistent lymphocytosis (PL); and 89 BLV-infected cattle with lymphoma. The circles/dots indicate the average proviral load detected in each stage, and the bar indicates the standard error. P-values were calculated by pairwise t-test using R version 2.10.1 (The R Foundation for Statistical Computing).

To characterize differences in BLV proviral load in the early and late stages of disease, we calculated BLV proviral copy numbers for 268 BLV-infected cattle in different stages of progression of EBL. We measured proviral load in 163 BLV-positive, healthy cattle, 16 BLV-infected cattle with PL, 89 BLV-infected cattle with lymphoma, and 117 BLV-free normal cattle by BLV-CoCoMo-qPCR (Figure 9). The proviral loads were significantly increased at the PL stage compared with the aleukemic stage (p = 0.0159) and were further increased at the lymphoma stage (p = 0.0052). No BLV was detected in the 117 BLV-free normal cattle. Thus, we were able to demonstrate that BLV proviral copy number increased with increasing severity of disease.

## Discussion

In this study, we describe the successful development of a highly specific, accurate, and sensitive method for the quantification of BLV proviral load from infected animals.

The BLV-CoCoMo-qPCR system is able to detect various BLV strains from a broad geographical origin, including Japan, Peru, Bolivia, Chile and the U.S.A. Although early studies on genetic variability of the BLV *env *gene identified very little variation among isolates [28], recent studies based on restriction fragment length polymorphism analysis and on analysis of the full-length BLV *env *gene have revealed at least seven different BLV genotypes in circulation worldwide [24,27,29]. This novel classification suggests that BLV divergence has increased worldwide. From a total of 356 BLV sequences in GenBank, we were able to obtain 52 distinct BLV LTR nucleotide sequences. This indicates that many BLV variants exist, and our results suggest that detection of all of these variants is possible using BLV-CoCoMo-qPCR. Thus, we have clearly demonstrated that the CoCoMo algorithm is a useful tool for designing degenerate primers corresponding to multiple BLV variants. In fact, Tong *et al. *[30] indicated that semi-nested or nested PCR assays with consensus-degenerate hybrid oligonucleotide primers for *Paramyxoviridae *could be developed to be either highly specific or more broadly inclusive, enabling targeting at the subfamily or genus level. Using this type of approach, there is a risk that the degeneracy of the CoCoMo primers could be too high, thereby reducing the concentration of primer specific to the target sequence and decreasing the assay's sensitivity. This issue did not arise in our study since BLV-CoCoMo-qPCR was highly sensitive and gave superior results to nested PCR amplification (Figure 6). In addition, the BLV-CoCoMo-qPCR system was very effective in detecting virus in BLV-infected cattle from a range of geographic locations (Table 3). The TaqMan probe was used to improve sensitivity and specificity and acts to counter any drawbacks associated with high degeneracy. It is important to note that the sequence of the BLV TaqMan probe, located between positions corresponding to the CoCoMo 6 and CoCoMo 81 primers, was completely conserved among the 52 BLV variants. The ELISA and immunodiffusion screening methods for BLV in cattle are also highly sensitive but they act by detecting antibodies against BLV, in contrast to the direct detection of integrated provirus by PCR [17]. The ELISA and immunodiffusion methods suffer from a high rate of false positives, and they are also ineffective in determining whether calves are infected since circulating maternal antibodies from BLV-infected dams can interfere with the assay. Reliable detection of BLV-infection in cattle therefore requires a high-sensitivity method for the detection of provirus. To this end, we developed BLV-CoCoMo-qPCR.

Several approaches were used to confirm the high specificity of BLV quantification using CoCoMo primers. First, the CoCoMo 6 and CoCoMo 81 primers yielded a single peak by melting curve analysis in cells infected with BLV, but did not amplify a product in uninfected cells or in the reagent-only negative control. Second, PCR amplification was detected in BLV-positive cattle, but was negative in all 120 BLV-negative cattle tested. Third, infectious molecular clones including several non-BLV retroviral LTRs were not amplified by our system.

A previous study [31] reported a method to quantify BLV provirus using real-time PCR. This method targeted the BLV *env *and *pol *genes, which are present at only one copy per provirus, and the primer annealing regions were potentially susceptible to mutation. The BLV LTR target of BLV-CoCoMo-qPCR is present at two copies per provirus, which contributes to the improved sensitivity of our assay. Indeed, using the quantitative PCR method described by Lew *et al. *[31], we could not detect provirus at less than 18 copies/10^5 ^cells, a concentration that was readily detectable by BLV-CoCoMo-qPCR (data not shown). Our method also has the advantage that the use of degenerate primers allows for the detection of BLV sequence variants, including those that arise from mutations.

The BLV-CoCoMo-qPCR method was also accurate. The provirus copy number obtained using real-time PCR with CoCoMo primers correlated closely with the result from serial dilution nested PCR. Because we aimed to use BLV-CoCoMo-qPCR to quantify cell-associated BLV provirus, we performed a parallel quantitation of the single-copy cellular gene *BoLA-DRA*. This measurement allowed adjustment for variations in amplification efficiency between samples. Using this strategy, we observed sufficient intra- and inter-assay reproducibility for the diagnosis of infected animals. The assay CV range was 0.0% to 20.5%, which was markedly better than that reported for quantification of HTLV-1 proviral load (8.2% to 31.4% [32] or 49% to 55% [33]). The high reproducibility of our assay enabled its use for the quantitation of proviral load during disease progression. The accuracy of BLV-CoCoMo-qPCR was also confirmed by sequencing analysis. We selected nine samples in which BLV provirus could be detected by BLV-CoCoMo-qPCR, but not by nested PCR, and sequenced the amplicons. Using this strategy, we confirmed that the nine samples were infected by BLV, and this highlights the ability of the BLV-CoCoMo-qPCR method to detect provirus in samples that were negative by nested PCR. In two of the nine samples, sequence mismatches were detected at the annealing region for the nested PCR primers, thereby suggesting an explanation for the failure of nested PCR to detect BLV in these samples.

The syncytia assay is a common strategy for detecting viable BLV virus particles [34]. However, this method requires cell culture, is time consuming and often difficult, and also has low sensitivity. We tested whether the proviral copy number obtained with our assay correlated with the syncytium formation assay, since this would suggest that our assay could be used for diagnosis at the BLV infection stage. Syncytia formation correlated strongly with a proviral load of over 10,000 copies/10^5 ^cells, as calculated by BLV-CoCoMo-qPCR. In BLV-infected cattle with a low proviral load detected by BLV-CoCoMo-qPCR, syncytia formation could hardly be detected. Thus, BLV-CoCoMo-qPCR appears to be capable of correctly determining the level of BLV infection in animals with low viral loads.

The pre-leukemic phase of BLV infection, called as PL, is characterized by the expansion of infected surface immunoglobulin M-positive B-cells with proviral insertion at multiple sites. On the other hand, a unique integration site is characteristic of malignant of malignant B-cells found in BLV-infected individuals after the onset of overt leukemia/lymphoma [13,23,35]. According to this model, proviral load should increase during disease progression but this has not been formally demonstrated. Using BLV-CoCoMo-qPCR, we were able to detect an increase in proviral load during disease progression. This result strongly suggests that proviral load may be an excellent indicator for monitoring the progression of disease, but may also be useful for implementing segregation programs to minimize BLV transmission. Previous experiments also identified host factors or genetic backgrounds that correlate with disease progression. For example, tumor-associated c143 antigens have been identified that are serine phosphorylated specifically in cattle with EBL, and genetic polymorphisms in cancer-associated genes such as p53 and tumor necrosis factor (TNF) have also been linked with EBL [35-41]. Proviral load may also be a valuable measure for identifying markers that influence progression to the lymphoma stage. Our assay may be valuable for estimating the effectiveness of vaccination and may also be capable of detecting changes in proviral load in BLV-infected cattle with the *TNF *and *BoLA *alleles that have previously been associated with resistance or susceptibility to BLV-induced lymphoma. Finally, since our assay detected all BLV variants, the CoCoMo algorithm appears to be a useful tool for designing degenerate primers for the quantification of proviral loads of other retroviruses, including HTLV and HIV-1.

## Conclusions

Using CoCoMo primers, we have developed a new quantitative real-time PCR method to measure the proviral load of known and novel BLV variants. Our method is highly specific, sensitive, quantitative and reproducible for detection of the BLV LTR region in infected animals. The method was effective in detecting BLV in cattle from a range of geographical locations, and detected BLV in a broader range of samples than the previously developed nested PCR. Finally, we have shown for the first time that the proviral load correlates well with the stage of disease progression.

## Methods

### Clinical samples, cell lines and DNA extraction

Blood samples were obtained from 117 healthy cattle with negative BLV serology, 163 BLV-infected cattle that were clinically and hematologically normal, 16 clinically normal BLV-infected cattle with PL, and 89 BLV-infected cattle with EBL. These cattle were all maintained in Japan. A further 116 cattle that were maintained in Bolivia, Peru, Chile and the U.S.A were included in the study. BLV-infected cattle were classified according to previously established criteria [42] and the genomic integrations of the BLV provirus. PBMCs were separated from blood by the method of Miyasaka and Trnka [43].

The BLV-infected B lymphoma cell line BLSC-KU-17 [44] was maintained in Dulbecco's modified Eagle's medium (Life Technologies Japan, Tokyo, Japan) supplemented with 10% heat-inactivated fetal calf serum (FCS) (Sigma Aldrich Chemie Gmbh, Steinem, Germany), penicillin and streptomycin. CC81, a cat cell line transformed by mouse sarcoma virus, was maintained in RPMI 1640 medium (Sigma-Aldrich Co. Ltd., Ayrshire, UK) supplemented with 10% heat-inactivated FCS, penicillin and streptomycin.

Genomic DNA was extracted from (a) whole blood by the DNA Wizard Genomic DNA purification Kit (Promega, Madison, WI), (b) PBMCs by the procedure described by Hughes *et al. *[45] and (c) 40 μl of whole blood spotted on FTA elute cards (Whatman, Tokyo, Japan), using standard procedures.

### Design of primers and probes

The 52 variants of individual BLV LTR sequences were selected from 356 BLV sequences in GenBank as shown in Additional files 1-3. The target sequences were subjected to a BLV LTR modified version of the CoCoMo-primer-design algorithm (http://www.geneknot.jp/cocomo; Endoh D, Mizutani T, Morikawa S Hamaguchi I, Sakai K, Takizawa K, Osa Y, Asakawa M, Kon Y, Hayashi M,: CoCoMo-Primers: a web server for designing degenerate primers for virus research. Submitted), which was developed for designing degenerate primers to detect multiple strains of viruses.

To detect specific PCR products, we used the TaqMan™probe system (Applied Biosystems, Tokyo, Japan), with probe sequences designed using Primer Express software, version 2.0 (Applied Biosystems).

### Plasmids

To obtain pBLV-LTR/SK, which included a full-length LTR from BLV, we used PCR with the primers BLV-LTR/XhoI (5'-CCCGCTCGAGTGTATGAAAGATCATGCCGA-3'; positions 1 to 20) and BLV-LTRR/BamHI (5'-CGGGATCCTGTTTGCCGGTCTCTCCTGG-3'; positions 511 to 531) and genomic DNA extracted from BLSC-KU-17 cells as a template. PCR products were cloned into pBluescript II SK (+) (Stratagene, La Jolla, CA). The numbering of nucleotides corresponds to positions in the sequences determined by Derse *et al. *[46]. To generate pBoLA-DRA/SK, which includes a full-length bovine *DRA *gene, we digested MR1 from the mammalian expression vector pCDM8 [47] with X*ba *I. The X*ba *I-X*ba *I fragment including the MR1 sequence was then subcloned into pBluescript II SK (+) (Stratagene). Additional clones used included a BLV infectious clone, pBLV-IF [34]; a HTLV-1 infectious clone, pK30 [48]; a HIV-1 infectious clone, pNL4-3 [49]; a SIV infectious clone, SIVmac239/WT [50]; a hybrid MMTV provirus plasmid [51]; a M-MLV infectious clone, pL-4 [52] and plasmids including pUC18 (Takara Bio Inc., Tokyo, Japan), pUC19 (Takara Bio Inc.), and pBR322 (Promega).

### Calculation of copy number by the serial dilution method

pBLV-LTR/SK and pBoLA-DRA/SK were digested with *Sca *I and purified using a Sephadex G-50 column (GE Healthcare Japan, Tokyo, Japan). The genomic DNA was digested with *Xho *I in the presence of 5 mM spermidine for 24 h. The samples were diluted to serial ten-fold dilutions with TE buffer [10 mM Tris-HCl (pH 8.0) with 1 mM ethylenediamine tetraacetic acid ] on-ice. To avoid DNA adsorption to the microtubes, super smooth processed tubes (BM4015 Platinum super polypropylene; BM bio, Tokyo, Japan) were used for the preparation of template for the standard curve.

Limiting dilutions were performed for linearized pBLV-LTR/SK, pBoLA-DRA/SK and genomic DNA. Detection of the BLV LTR gene and the *BoLA-DRA *gene from plasmid DNA was performed by real-time PCR with the CoCoMo primers 6 and 81, or the primers DRA643 and DRA734, respectively. The BLV LTR gene was also detected in genomic DNA using nested PCR. At the dilution point at which amplification products were unable to be detected, PCRs were repeated 10 times and the frequency of negative results was calculated (f(x = 0)). The copy numbers of the target genes were calculated according to a Poisson distribution model: λ = -log_e_(f(x = 0)), where λ = average copy number of the target gene.

### PCR conditions for candidate CoCoMo primer sets for the amplification of the BLV LTR

Touch-down PCR amplifications were carried out in a 20 μl volume of 1 × buffer for rTaq DNA Polymerase (TOYOBO, Tokyo, Japan) containing 1.0 unit rTaq, 0.2 mM dNTPs, 1.0 mM MgCl_2_, 500 nM of forward and reverse primers, and 1 μl of 1:1000 diluted BLV LTR amplicon, which had been amplified from genomic DNA by nested PCR. PCR amplification was performed with a TGRADIENT thermocycler (Biometra, Göttingen, Germany) according to the following program: an initial denaturation at 95°C for 10 min, followed by 40 cycles of 15 s at 95°C, 10 s at 60°C to 52°C (annealing temperature was gradually decreased from 60°C to 52°C, by 0.2°C every three cycles) and 10 s at 72°C. Five μl of PCR products was used for 2% agarose gel electrophoresis and amplification products were detected by ethidium bromide staining.

### Melting curve analysis for evaluating PCR specificity

PCR amplifications took place in a total volume of 20 μl of 1 × LightCycler FastStart DNA Master SYBR Green I (Roche Diagnostics GmbH, Basel, Switzerland) containing 500 nM of each of the CoCoMo primers, 3 mM of MgCl_2_, and 30 ng of genomic DNA. PCR amplifications were performed with a Light Cycler 2.0 (Roche Diagnostics GmbH) according to the following program: an initial denaturation at 95°C for 10 min, followed by 75 cycles of 15 s at 95°C, 5 s at 65°C, and 9 s at 72°C. The melting process was monitored by fluorescence of the DNA-binding SYBR Green I dye for the detection of double-stranded DNA.

### Detection of BLV LTR by nested PCR

The first PCR amplification was done using the primers BLTRF-YR (5'-TGTATGAAAGATCATGYCGRC-3' LTR 1-21) and BLTRR (5'- AATTGTTTGCCGGTCTCTC-3' LTR 515-533). The amplifications were carried out in a total volume of 20 μl of 1 × buffer for rTaq DNA Polymerase (TOYOBO) containing 250 nM of BLTRF-YR primer and BLTRR primer, 0.5 units of rTaq polymerase, 0.2 mM dNTPs, 2.5 mM MgCl_2_, and 30 ng of template DNA. PCR amplification was performed with a TGRADIENT thermocycler (Biometra) according to the following program: an initial denaturation at 94°C for 2 min, followed by 35 cycles of 30 sec at 94°C, 30 sec at 58°C and 30 s at 72°C, and a final cycle of 5 min at 72°C.

The second set of PCR amplifications was performed in a total volume of 20 μl of 1 × buffer for rTaq DNA Polymerase (TOYOBO) containing 250 nM of 256 primer and 453 primer (see below), 0.5 units of rTaq polymerase, 0.2 mM of dNTPs, 2.5 mM of MgCl_2_, and 1 μl of first-round PCR product. The oligonucleotide sequences used in the second PCR were 256 (5'-GAGCTCTCTTGCTCCCGAGAC-3', LTR 256-276) and 453 (5'-GAAACAAACGCGGGTGCAAGCCAG-3', LTR 430-454), and have been described previously [23]. PCR amplification was performed with a TGRADIENT thermocycler (Biometra) according to the following program: an initial denaturation at 94°C for 2 min, followed by 35 cycles of 30 s at 94°C, 30 s at 58°C and 30 s at 72°C, and a final cycle of 5 min at 72°C. Five μl of PCR products was used for 2% agarose gel electrophoresis and the PCR products were detected by ethidium bromide staining.

### Amplification, Cloning, and DNA sequencing of BLV LTR regions

To analyze the nucleotide sequence of samples that were positive by BLV-CoCoMo-qPCR but negative by nested PCR, genomic DNA from BLV-infected cattle was subjected to amplification by PCR using MightyAmp DNA polymerase Ver.2 (TAKARA), KOD plus Neo (TOYOBO), and TaqMan Universal Master Mix II system (AB). BLV LTR specific oligonucleotide primers BLTR56F (5'-AACGTCAGCTGCCAGAAA-3'), BLTR134F (5'-AAAATCCACACCCTGAGCTG-3'), CoCoMo6, CoCoMo81, and BLTR544R (5'- ACGAGCCCCCAATTGTTT-3') were designed by reference to the LTR regions of BLV proviral sequences. The PCR products were subcloned into pGEM-T Easy vector (Promega) by TA cloning and the nucleotide sequence was determined by cycle sequencing using standard procedures.

### Syncytium formation assay

The assay was performed according to a previously described procedure [15,22]. CC81 cells were grown for 72 h in a 6-cm-diameter dish and incubated with 4 × 10^5 ^PBMC from BLV-infected cattle in RPMI 1640 medium. Cells were fixed in May-Grunwald solution for 2 min and stained with Giemsa solution for 15 min. After washing with water, cells were examined under a light microscope. Cells containing more than five nuclei were counted as syncytia.

### Statistical analysis

Statistical analysis was conducted using R 2.10.1 statistical computing software. For multiple testing, a pairwise t test was used for calculating pairwise comparisons between group levels with corrections. P-values of less than 0.05 were considered significant. Regression analysis was used to examine the correlation between the serial dilution method and the BLV-CoCoMo-qPCR method, and between the syncytium count and the provirus copy number.

## Competing interests

The authors declare that they have no competing interests.

## Authors' contributions

MJ participated in all experiments, analyzed data and drafted the manuscript. ST carried out experiments, participated in the experimental design, analyzed data, and helped to draft the manuscript. KM participated in some experiments and sample collection. DE participated in the design of the CoCoMo primers. YA conceived the study, participated in experiments, participated in experimental design, coordinated experiments, and drafted the manuscript. All authors read and approved the final manuscript.

## Supplementary Material

Additional file 1 **From the BLV sequences, 102 LTR sequences were selected based on GenBank annotations**. We collected whole nucleotide sequence-data from GenBank (356 data, on 30th April, 2009). From the BLV sequences, we selected 102 LTR sequences based on GenBank annotations. Click here for file

Additional file 2 **LTR-network**. From the 102 LTR sequences, we selected 85 sequences that were of sufficient sizes ( > 400 bp) to determine homologies, and assigned the sequences to major BLV LTR groups based on homology using a graphical approach with Pajek graphical software. Click here for file

Additional file 3 **Selection of representative BLV LTR sequences**. In the 85 LTR sequence data, we classified homologous sequence groups and selected one sequence each group. Resultantly, 52 sequences, which were representing sequence repertoire of BLV LTR sequence, were selected as the target-set for primer-design. Click here for file
