# Supplementary figures and images for: BLV-CoCoMo-qPCR: Quantitation of bovine leukemia virus proviral load using the CoCoMo algorithm
Source: Retrovirology. 2010 Nov 2;7:91. doi: 10.1186/1742-4690-7-91 (PMC2988707; doi:10.1186/1742-4690-7-91)

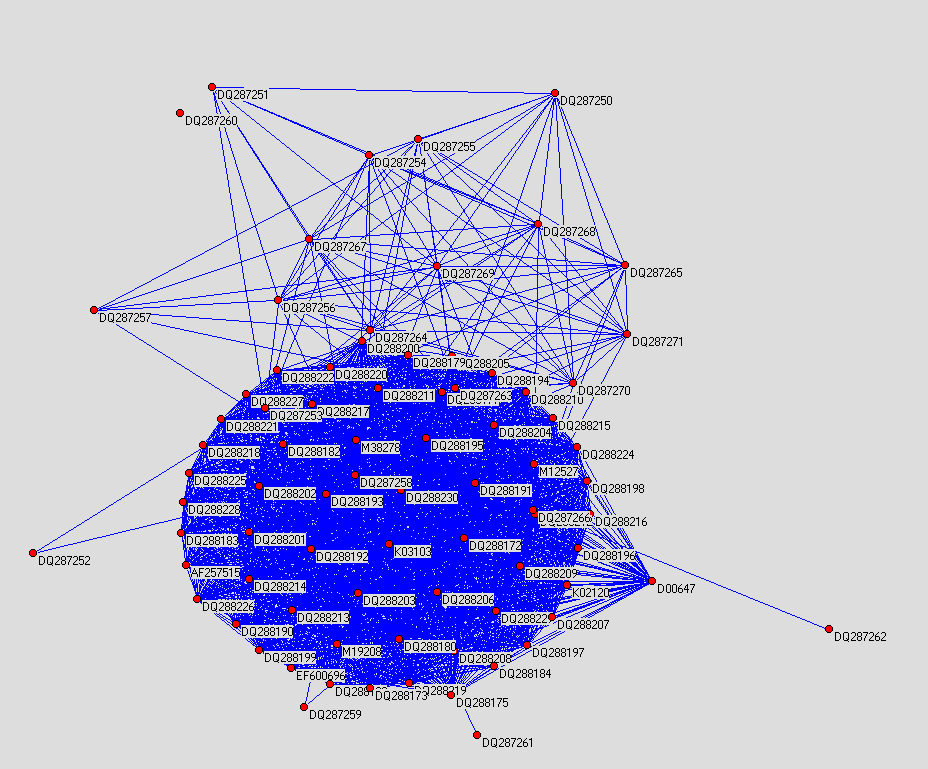

Supplement: Additional file 2 — LTR-network. From the 102 LTR sequences, we selected 85 sequences that were of sufficient sizes ( > 400 bp) to determine homologies, and assigned the sequences to major BLV LTR groups based on homology using a graphical approach with Pajek graphical software. [file 1742-4690-7-91-S2.PNG]
